# How Online Communities of People With Long-Term Conditions Function and Evolve: Network Analysis of the Structure and Dynamics of the Asthma UK and British Lung Foundation Online Communities

**DOI:** 10.2196/jmir.9952

**Published:** 2018-07-11

**Authors:** Sagar Joglekar, Nishanth Sastry, Neil S Coulson, Stephanie JC Taylor, Anita Patel, Robbie Duschinsky, Amrutha Anand, Matt Jameson Evans, Chris J Griffiths, Aziz Sheikh, Pietro Panzarasa, Anna De Simoni

**Affiliations:** ^1^ Department of Informatics King's College London London United Kingdom; ^2^ School of Medicine University of Nottingham Nottingham United Kingdom; ^3^ Asthma UK Centre for Applied Research Barts Institute of Population Health Sciences Queen Mary University of London London United Kingdom; ^4^ Primary Care Unit Department of Public Health and Primary Care University of Cambridge Cambridge United Kingdom; ^5^ HealthUnlocked London United Kingdom; ^6^ Asthma UK Centre for Applied Research Usher Institute of Population Sciences and Informatics University of Edinburgh Edinburgh United Kingdom; ^7^ School of Business and Management Queen Mary University of London London United Kingdom

**Keywords:** asthma, chronic obstructive pulmonary disease, COPD, network analysis, online community, online forums, superusers, self-management, digital health social network

## Abstract

**Background:**

Self-management support can improve health and reduce health care utilization by people with long-term conditions. Online communities for people with long-term conditions have the potential to influence health, usage of health care resources, and facilitate illness self-management. Only recently, however, has evidence been reported on how such communities function and evolve, and how they support self-management of long-term conditions in practice.

**Objective:**

The aim of this study is to gain a better understanding of the mechanisms underlying online self-management support systems by analyzing the structure and dynamics of the networks connecting users who write posts over time.

**Methods:**

We conducted a longitudinal network analysis of anonymized data from 2 patients’ online communities from the United Kingdom: the Asthma UK and the British Lung Foundation (BLF) communities in 2006-2016 and 2012-2016, respectively.

**Results:**

The number of users and activity grew steadily over time, reaching 3345 users and 32,780 posts in the Asthma UK community, and 19,837 users and 875,151 posts in the BLF community. People who wrote posts in the Asthma UK forum tended to write at an interval of 1-20 days and six months, while those in the BLF community wrote at an interval of two days. In both communities, most pairs of users could reach one another either directly or indirectly through other users. Those who wrote a disproportionally large number of posts (the superusers) represented 1% of the overall population of both Asthma UK and BLF communities and accounted for 32% and 49% of the posts, respectively. Sensitivity analysis showed that the removal of superusers would cause the communities to collapse. Thus, interactions were held together by very few superusers, who posted frequently and regularly, 65% of them at least every 1.7 days in the BLF community and 70% every 3.1 days in the Asthma UK community. Their posting activity indirectly facilitated tie formation between other users. Superusers were a constantly available resource, with a mean of 80 and 20 superusers active at any one time in the BLF and Asthma UK communities, respectively. Over time, the more active users became, the more likely they were to reply to other users’ posts rather than to write new ones, shifting from a help-seeking to a help-giving role. This might suggest that superusers were more likely to provide than to seek advice.

**Conclusions:**

In this study, we uncover key structural properties related to the way users interact and sustain online health communities. Superusers’ engagement plays a fundamental sustaining role and deserves research attention. Further studies are needed to explore network determinants of the effectiveness of online engagement concerning health-related outcomes. In resource-constrained health care systems, scaling up online communities may offer a potentially accessible, wide-reaching and cost-effective intervention facilitating greater levels of self-management.

## Introduction

### Background

Online communities have the potential to influence health and health care. Recent studies have suggested that the participation of people with long-term conditions (LTCs) in online communities (1) improves illness self-management [1], (2) produces positive health-related outcomes [2-4], (3) facilitates shared decision-making with health care professionals [5,6], and (4) may even reduce mortality [7].

There is also evidence that self-management support interventions can reduce health service utilization [8,9].

Online communities have experienced an upsurge in popularity among people with chronic respiratory conditions such as cystic fibrosis [10], asthma [11], pulmonary hypertension [12] and chronic obstructive pulmonary disease (COPD) [13]. More than 15 million people in England suffer from a long-term condition or disability, and they account for at least 50 percent of all general practitioner appointments [14,15]. Thus, assessing how these online communities function and evolve can have important implications for health care provision.

This form of “user-led self-management” of LTCs bears similarities with the “expert patient” model, an approach to self-management of LTCs produced by the United Kingdom (UK) Department of Health in 2001 [16]. Evidence of the effectiveness of conventional off-line self-management programs based on the expert patient model, though, has been weak [17]. Clinic-based self-management programs often failed because of: (1) lack of awareness and engagement among patients and staff, (2) failure to consider low health literacy or cultural norms, (3) lack of attention to the need for family and social support, and (4) a fragmented approach to the provision of health and social care [18]. Although online health communities can be seen as an extension of the expert patient model, network effects, in addition to the online disinhibition effect [19], make them a distinct and unique complex intervention mechanism.

On average, one in four people with an LTC who use the Internet tries to engage online with others with similar health-related concerns [20]. In particular, it has been suggested that the value of participating in an online community lies in the possibility of gaining access to a range of people and resources quickly, easily [21], and anonymously [4], as well as obtaining tailored information and emotional support [1,22-26]. However, most of this evidence comes from qualitative studies [1,27], whereas only recent years have witnessed an increasing interest in quantitative assessments of online communities as intervention mechanisms [28-33]. Recent studies have been concerned with the users’ unequal contributions and engagement patterns, and with the role of superusers. However, the contribution of superusers to the sustainability of online health communities and their structural properties remains mostly unclear.

The potential future integration of online health support systems with formal health care provision should be underpinned by a better understanding of how they are used and by evidence of their effectiveness. Indeed, as suggested by the Medical Research Council [34], integrating online support systems with the more traditional health care provision would require the identification and comparative assessment of potential alternative intervention mechanisms.

An expanding body of literature concerned with social network analysis has examined the structural patterns of relations among interacting actors and the social mechanisms that enable them to gain access to valuable resources [35]. There is also increasing evidence that network approaches can be applied to understanding the users’ “expertise” [36], their interactions, and network effects on health-related outcomes in online health communities [37,38]. Uncovering the mechanisms underlying the formation of successful social networks requires a study of how online connections among people, namely the social ties or links, emerge and evolve, and how groups of individuals gradually grow in membership and become interconnected with one another. These processes of tie creation and group formation in online patients’ communities are still mostly unexplored [1].

In this study, we performed a network analysis of the structure and dynamics of two online communities of people with LTCs. We chose the Asthma UK and the British Lung Foundation (BLF) communities as an exemplar of such communities because their users typically suffer from chronic respiratory conditions. In particular, while Asthma UK users typically suffer from a respiratory condition characterized by variable and recurring symptoms, BLF users represent a more heterogeneous population of participants affected by different diseases linked to chronic symptoms of breathlessness (eg, COPD, pulmonary fibrosis, cystic fibrosis, and lung cancer).

**Research questions.**

1. What is the network structure of online communities for people with long-term conditions, and how do they function and evolve over time?
2. Does posting activity follow a time pattern?
3. Are there (a minority of) users with a special role in maintaining integration and cohesion of the community?
4. Do superusers write their posts uniformly over time or do they produce peaks of activity separated by periods of inactivity?
5. For how long do superusers remain active in an online community?
6. Are superusers help-seekers or help-givers?
7. Do superusers preferentially write posts to each other or to users who write relatively few posts?
8. Is there any association between users’ interaction patterns and their potential for enhancing peer self-management support in the community?
9. Do online health communities function and evolve in the same way as other real-world complex systems?

We aimed to uncover and understand how these communities function and evolve, and the role that some users have in maintaining integration and cohesion (see Textbox 1 for research questions). Ultimately, this study provides evidence for gauging the effectiveness of different interaction patterns and the users’ structural positions and their potential for enhancing and sustaining health online communities as scalable self-management support interventions.

## Methods

### Data Collection

Data were collected by HealthUnlocked [39], the online platform provider of the Asthma UK and BLF communities. Registered users can choose to either write posts publicly or send private posts to one another. In the latter case, posts are shared between 2 users only, whereas when posts are written publicly, a large number of users can become connected through threads of posts. Only posts that were shared publicly were collected and analyzed. For this study, user identifiers (IDs) were anonymized by HealthUnlocked, and no demographic information was collected. The data sets included posts and their metadata (ie, the anonymized user ID numbers), user roles (eg, user, administrator, or moderator), date of posting, the hierarchical level of the post within the corresponding thread, and the dates in which the users joined and left the community. Both communities were moderated, and HealthUnlocked moderators (identified through metadata linked to posts) were included in the analysis to assess their contribution and compare it with other users. Online communities on the HealthUnlocked platform benefit from additional functionalities compared to other online forums, such as built-in patient groups that moderate the content. In particular, the content accessed by users is tailored to their interests, and profiles highlight users’ condition, chosen community, medications and treatments they use or find interesting. No data were collected on participants’ characteristics, though only people declaring themselves to be older than 16 years were permitted to create an account and take part in the online communities.

### Data Analysis

We looked at the number of users, the number of posts and connections per user and posting frequency. A connection (ie, a tie, link, or edge) was established from one user to another when the former replied to a post by the latter (see Textbox 2 for network analysis terminology). The pattern of connections generated over time through the cumulative number of posts and replies was examined. We were interested not just in the number of posts and responses but in who responded to whom, and when. To this end, we used social network analysis [40] to visualize and study the structure of the relationships between users. Both visualization and analysis were conducted using the Gephi software. The network analysis was carried out through additional custom computer code in python. Descriptive analysis of the networks (ie, number of users, posts, and posting frequency) were calculated using the Pandas library, an open source library providing data structures and analysis tools for the Python programming language.

As a result of the small percentage of users who wrote posts to a disproportionally high number of users, the users’ activity showed long-tailed distributions. Therefore, our analysis was based not only on means and standard deviations but also on medians.

To uncover time patterns in posting activity, we used Fourier transforms of the time series of the users’ activity [46], a known method used for the analysis of signals. Through Fourier transforms, we identified the frequency components, called harmonics, that together made up the posting activity stream. In other words, we regarded the posting activity over the entire observation period in both communities as a complex signal and identified the frequency components that made up such a signal. This analysis was performed using custom code in Scipy, a Python-based scientific computing library.

The “rich-club” coefficient is a metric designed to measure the extent to which well-connected users tend to connect with one another to a higher degree than expected by chance [43]. To this end, for each value *k* of a node’s degree (ie, the number of other users a given user is connected with), we computed the ratio between the number of actual connections between nodes with degree *k* or larger and the total possible number of such connections [47]. We then divided this ratio by the one obtained on a corresponding random network with the same number of nodes and degree distribution (ie, the probability distribution of the degrees over the whole network) as the real network, but in which links were randomly reshuffled between nodes. Thus, the rich-club coefficients may take values lower or higher than 1, depending on whether the real network has a higher or lower tendency to coalesce into rich clubs than randomly expected. In particular, networks that display a high rich-club coefficient (ie, greater than 1) are also said to show a “rich-club effect,” namely the tendency to organise into a hierarchical structure in which highly connected nodes preferentially create tightly knit groups with one another, thus generating exclusive clubs of (topologically) rich nodes, as illustrated in previous work [48].

In our study, superusers were defined according to their cumulative activity over the entire observation period. In total, we identified 400 superusers. To uncover how many superusers were active within each week, we detected how many unique users, among the 400 identified over the entire period, were active within that time window.

**Figure 1:**
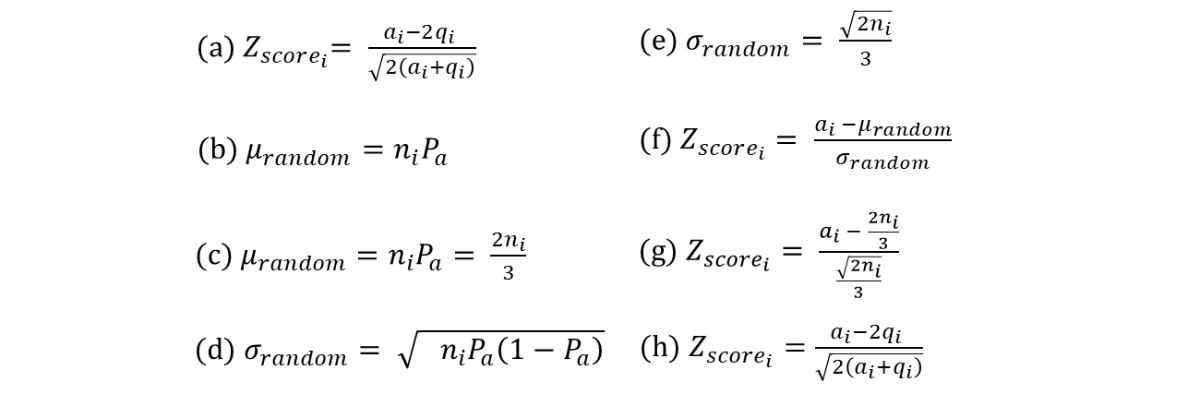
The *z*-score used as a proxy for users’ expertise.

Following Zhang et al [36], the “*z*-score” was used as a proxy for users’ expertise. According to this measure, replying to many questions suggests one’s expertise, while asking questions indicates lack of expertise. In our analysis, we treated anyone starting a thread as a help-seeker, and anyone commenting on the thread as a help-giver [36]. Accordingly, the proposed *z*-score aims to capture the combined help-seeking and help-giving patterns. To this end, for each user, we measured how many standard deviations the observed total number of the user’s help-giving posts lies above or below the expected number of help-giving posts for the whole system. We extended the approach proposed by Zhang et al by empirically assessing the probability of posting and answering a question across all users over the entire observation period. In the BLF community, we found that the probability of answering is *P*_a_=2/3, while the probability of posting is *P*_q_=1/3. We assumed a Bernoulli process of posting an answer or a question to the forum, with probabilities defined as above. The *z*-score for a given user *i* was calculated according to equation (a) in Figure 1, where *a_i_* refers to the total number of answers user *i* posted to the forum, *q_i_* is the total number of questions user *i* asked in the forum, and *n_i_*=*a_i_* +*q_i_* is the total number of messages posted by user *i*.

**Network analysis terminology.**

- Degree: the number of connections a user has established with other users through posts
- Ego(-centred) network: the subset of connections linking a focal user—“ego”—directly to other users—“alters”—and connections linking these alters with each other
- Largest component: the network component (see below) with the largest number of members.
- Network Component: a subset of the network in which all members are directly or indirectly connected with one another (ie, all pairs of nodes in the subset are reachable through at least one tie) [41,42]. Each isolated user can be regarded as a separate component
- Node: individual user in an online community
- Rich-club coefficient: the degree to which highly connected users preferentially connect to each other to a higher degree than would be expected by chance. In a community with a rich-club coefficient higher than 1, users who post to many others preferentially communicate with each other, thus forming rich clubs. Conversely, in a community with a rich-club coefficient lower than 1, users who post to many others preferentially communicate with those who post to few others, thus generating an anti-rich-club behavior [43]
- Root post: the initial post in a thread of posts
- Superusers: top 1% of users characterised by the largest number of posts written in the community over the entire observation period [44]
- Tie, link, edge: online connection from a user to another, created when the former writes a post to the latter
- Triad: a group of 3 users—nodes *i*, *j*, and *u* —forming a path of length 2 (ie, node *i* is connected to node *j*, and node *j* is connected to node *u*). When node *i* is also connected to node *u*, the path is closed, forming a loop of length 3 or a triangle
- *z*-score: a measure of users’ expertise, capturing the users’ combined “help-seeking” and “help-giving” patterns. If a user writes help-seeking and help-giving posts equally often, then the user’s *z*-score would be equal to zero. Conversely, if a user writes more (or fewer) help-giving posts than help-seeking ones, then the *z*-score would be positive (or negative) [36,45]

To obtain *Z*_score_i__, let us define a random user that posts the same total number of messages *n*_random_ to the forum as user *i* (ie, *n*_random_=*n_i_*). We would expect this random user to post an average number of answers to the forum given by equation (b). Plugging in the value of *P*_a_=2/3, we obtained equation (c). Similarly, we would expect the random user to post answers with a standard deviation given by equation (d). Plugging in the value of *P*_a_=2/3, we obtained equation (e). To measure how many standard deviations above or below the expected random value a user *i* lies, we then computed *Z*_score_i__ according to equation (f). Plugging in the values of μ_random_ and σ_random_, we obtained equation (g). Finally, by substituting *n_i_*=*a_i_* +*q_i_*, we obtained equation (h).

### Ethical Considerations

Permission to research was obtained from Asthma UK and the BLF before starting the study. The research protocol was examined, and permission to research was obtained from Asthma UK, BLF charities and HealthUnlocked. The study was examined by the institutional Research Ethics board at Queen Mary University of London and was exempt from full review.

## Results

### Descriptions of Data Sets

The data sets span, respectively, 10 years for the Asthma UK and 4 years for the BLF communities (see Table 1).

**Figure 2:**
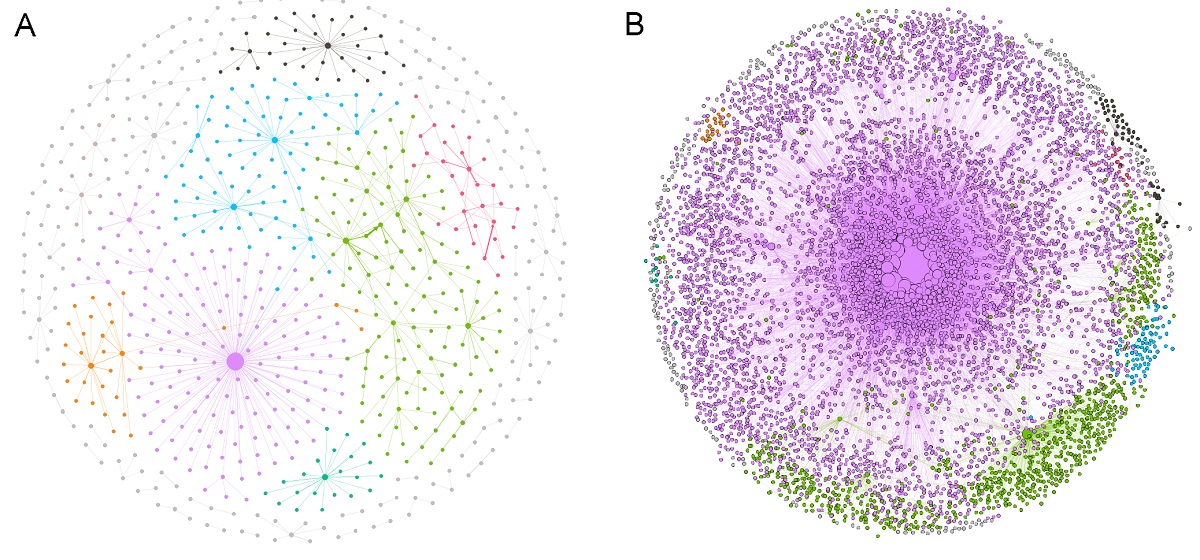
Cumulative networks across the time span analyzed. Each node represents a user. (A) Asthma UK users (around 1000); (B) British Lung Foundation users (around 8000). The coloring of nodes is based on modularity membership and the size of the node is proportional to its degree (ie, the number of connections with other users).

Despite the shorter time span, as a result of the larger number of users, the number of posts in the BLF community was higher than in Asthma UK, namely 875,151 compared to 32,780 respectively. Moreover, BLF users wrote a higher number of posts per user and were connected with a higher number of other users when compared with people in the Asthma UK forum (see Figure 2). In both communities, 60%-70% of registered users wrote no posts (ie, they were lurkers). Users who wrote more than one post contributed with a median of 8 (range 2-8947) and 5 (range 2-1068) posts in the BLF and Asthma UK communities, respectively.

The number of official moderators among the highly active users was negligible; there were no moderators in the top 5% contributors to BLF and only 2 in the top 5% for Asthma UK. Thus, our network analysis predominantly reflects content originated from registered users.

When classified according to posting activity (ie, number of posts written to the forum), the top 5% users contributed to a substantial proportion of all posts: 58% and 79% in the Asthma UK and BLF communities, respectively. Superusers were those who made a high number of connections with other users in both Asthma UK and BLF communities (see nodes of large size in Figure 2). Asthma UK superusers made a lower number of connections than BLF ones. The posting activity of these superusers will be analyzed in more detail in subsequent sections.

**Table 1:** Description of the Asthma UK and British Lung Foundation data sets.

Variables	Asthma UK	British Lung Foundation
Data set time span (mm/dd/yyyy)	02/03/2006-06/09/2016	13/04/2012-06/09/2016
Total time (weeks)	548	230
Total number of posts, n	32,780	875,151
Number of posts with reply, n (%)	28,615 (87.3)	815,184 (93.1)
Number of posts with no reply, n (%)	4165 (12.7)	59,967 (6.9)
Total number of users, n	3345	19,837
Users who wrote ≥1 post, n (%)	1053 (31.5)	7814 (39.4)
Users who wrote 1 post, n (%)	331 (31.4)	1186 (15.2)
Users who wrote >1 post, n (%)	722 (68.6)	6628 (84.8)
Registered users who never posted (ie, lurkers), n (%)	2292 (68.5)	12,023 (60.6)
Number of posts per user, mean (SD)	14.2 (55.0)	66.9 (75.1)
Number of posts per users who posted >1, median (range)	5.1 (2-1068)	8.0 (2-8947)
Number of posts per users who posted >1, mean (SD)	20.4 (65.6)	88.1 (458.6)
Posts contributed by top 1% superusers, n (%)	10,457 (31.9)	426,198 (48.7)
Number of connections per user, mean (SD)	2.1 (5.9)	17.6 (69.0)
Number of connections per user, median (SD)	1.0 (5.9)	1.0 (69.0)
Number of connections per top 1% superuser, mean (SD)	10.5 (16.5)	141.0 (174.0)
Number of connections per top 1% superuser, median (SD)	7.0 (16.5)	70.0 (174.0)

**Figure 3:**
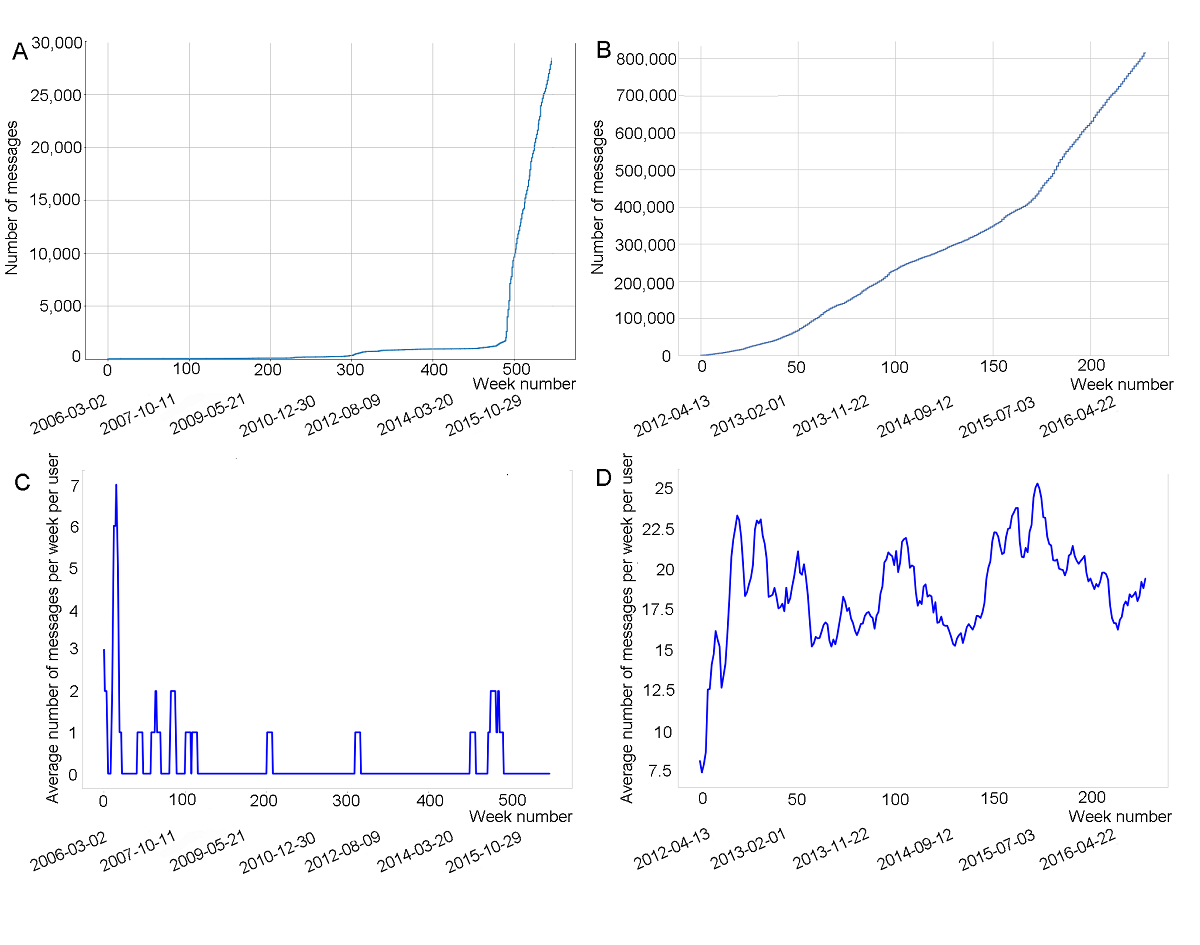
Cumulative distributions of the number of posts as a function of time (weeks) within the Asthma UK (A) and the British Lung Foundation (B) communities. Calendars dates are reported below week numbers. Panels C and D illustrate the average number of posts per user per week within Asthma UK and British Lung Foundation, respectively.

### Posting Activity

The cumulative number of messages posted grew uniformly over time in the BLF community. By contrast, in 2015, the Asthma UK forum witnessed a substantial increase in posting activity, at a time coinciding with its move to the HealthUnlocked platform (see Figure 3A and B). This increase in activity can be attributed to the online community functionalities offered by HealthUnlocked, as described in the Methods.

**Figure 4:**
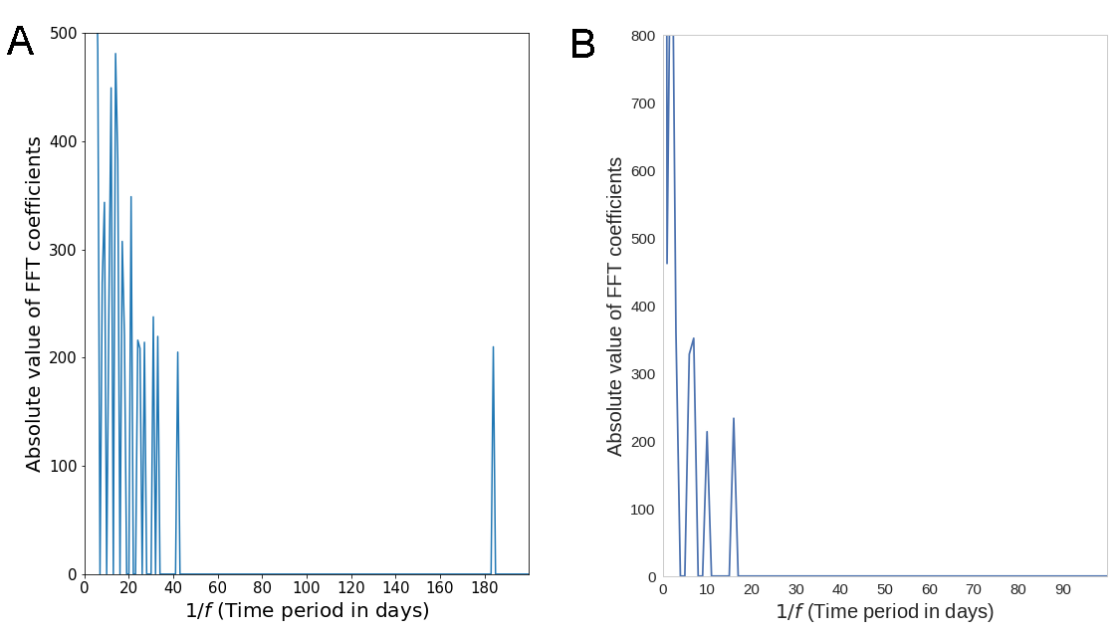
Periodicity of posting activity in Asthma UK (A) and the British Lung Foundation (B), measured through the Fast Fourier Transform (FFT). The component frequencies are denoted by *f* and are inverted to produce time period in days.

The number of posts per user per week oscillated around a decreasing and an increasing trend (Figure 2C and D), while at the same time the number of posts always went up over the study period (Figure 1A and B). This suggests that there were intervals of time during which the rate of increase in new users was larger than the rate of increase in total posts. Moreover, in the Asthma UK forum users wrote according to two time patterns—they posted at an interval of 1-20 days or 6 months (Figure 4A), while those in the BLF community at an interval of 2 days (Figure 4B).

As more users joined the communities and connected to one another through online posts, distinct groups of connected users started to emerge. These groups, called network components (see Textbox 2), have fundamental implications for the effectiveness of processes of network dynamics such as information diffusion [49]. In a relatively short period, both communities underwent the formation of the “largest component” of connected users, namely a connected subset of users whose size increasingly outgrew the size of all other components (see Figures 1 and 4, and Multimedia Appendices 1 and 2). The largest connected components in both communities included 60%-100% of users.

**Figure 5:**
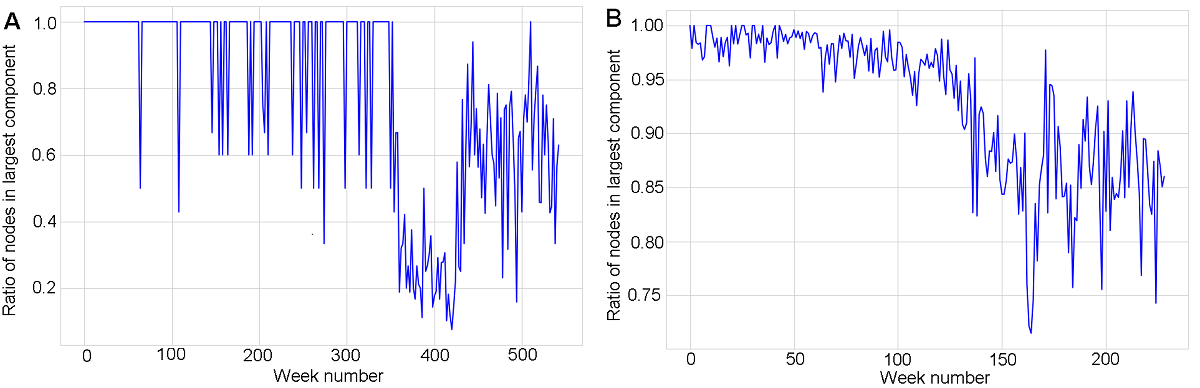
Fraction of users that are part of the largest component as a function of time (weeks) for Asthma UK (A) and the British Lung Foundation (B).

Figure 5 suggests that, as time went by, the number of forum participants and their posting activity increased, and the proportion of users who were part of the largest components decreased. This finding was expected because the number of posts also rose exponentially, yet at times at a lower rate than the one at which new users joined the communities (see Figure 1C and D). It, therefore, became more difficult for the network to self-organize into a connected component that would include 100% of the users. Figure 5A also shows that around week 450, when the forum moved to the HealthUnlocked platform, a larger fraction of users began to join the largest connected component, thus highlighting the role that the new online platform played in strengthening the connectedness of the network (see also Figure 3A and B).

### Superusers

Superusers represented a small minority (ie, 1%-5%) within both communities but were responsible for a high proportion of the posting activity and the functioning of the communities.

### Superusers’ Role

**Figure 6:**
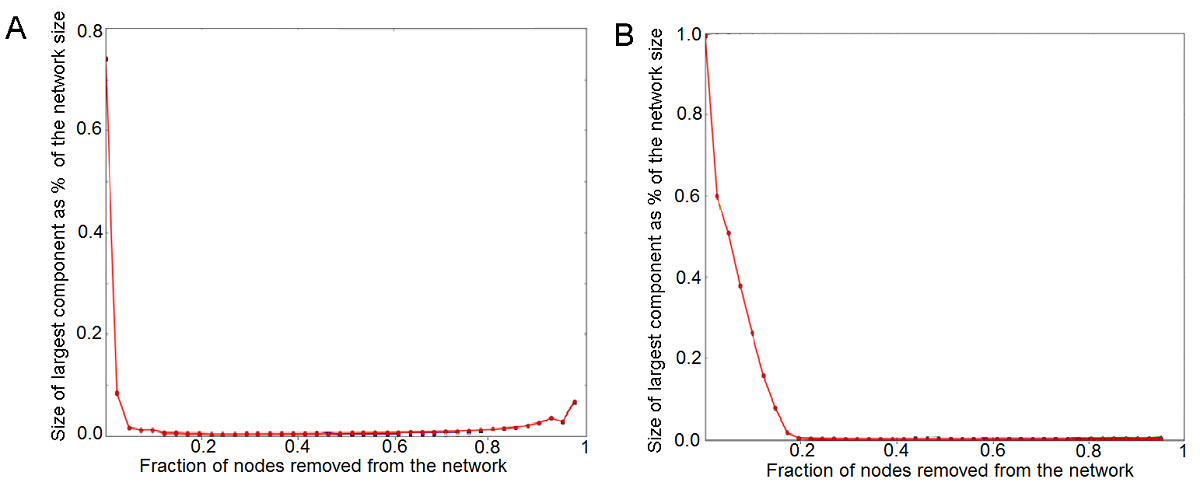
Sensitivity analysis: targeted removal of nodes (users) starting from the most connected ones within Asthma UK (A) and the British Lung Foundation (B).

Sensitivity analysis showed that the removal of users with the largest number of connections caused the largest component to collapse (see Figure 6), thus suggesting that both communities and lines of communication within them were held together precisely by these highly connected users. In online communities, the existence of groups of highly connected users is critical for information diffusion [50].

Figure 6 suggests that it only takes the removal of the top 5% users by degree of connectivity for the largest connected component to collapse to 10% and 50% of its original size in the Asthma UK and BLF communities, respectively. This corresponds to the removal of about 50 and 400 users in the 2 communities, respectively. These results shed light on how many superusers are needed to sustain discussions and to serve the needs of users in large communities of people with LTCs.

### Superusers and the Rich-Club Effect

**Figure 7:**
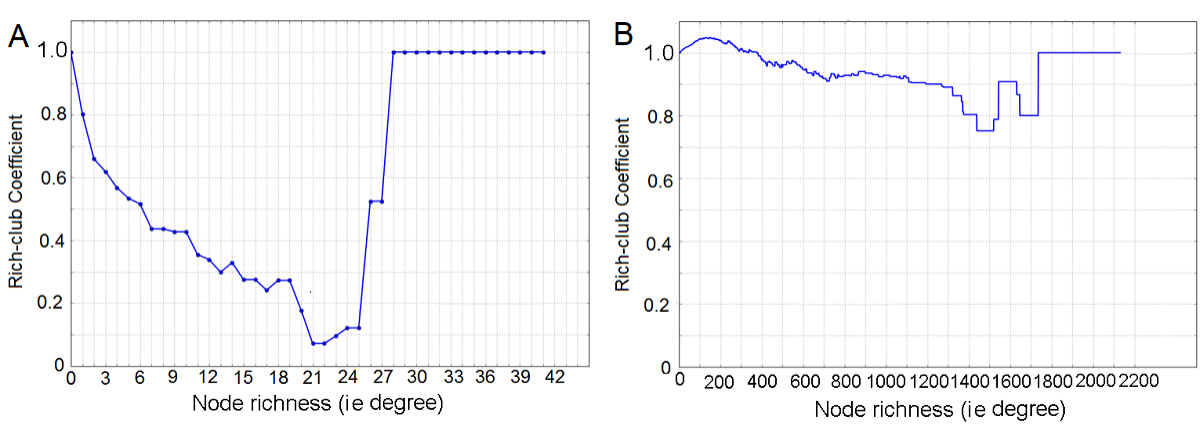
Rich-club coefficient as a function of the richness parameter (ie, users’ degree).

Both Asthma UK and BLF communities were characterized by a low rich-club coefficient, which was consistently lower than 1 (see Figure 7). This anti-rich-club behavior, namely the tendency to run counter to the formation of a rich club, suggests that in both communities highly connected superusers preferentially communicated with poorly connected ones or, alternatively, that superusers tended to avoid each other and instead communicated with those who were only connected with very few others.

Anti-rich-club behavior may suggest competition between superusers or merely the organization of the communities into groups of users characterized by different degrees of “expertise” or commitment: one group including the few committed experts and another including the vast majority of those seeking information when needed. It would, therefore, come as no surprise if the former were to communicate with the latter to a greater extent than randomly expected. We shall investigate this hypothesis further below.

### Involvement of Superusers Over Time

We have shown that the connectedness of both communities depends crucially on the presence and activities of superusers, who committed a significant amount of their time to writing posts and targeting new users. We now look at whether their activity was concentrated in relatively short periods of time or instead it was uniformly distributed over time. How superusers’ involvement is distributed over time may have fundamental implications for the cohesion of the whole system precisely in light of the role these users play.

**Figure 8:**
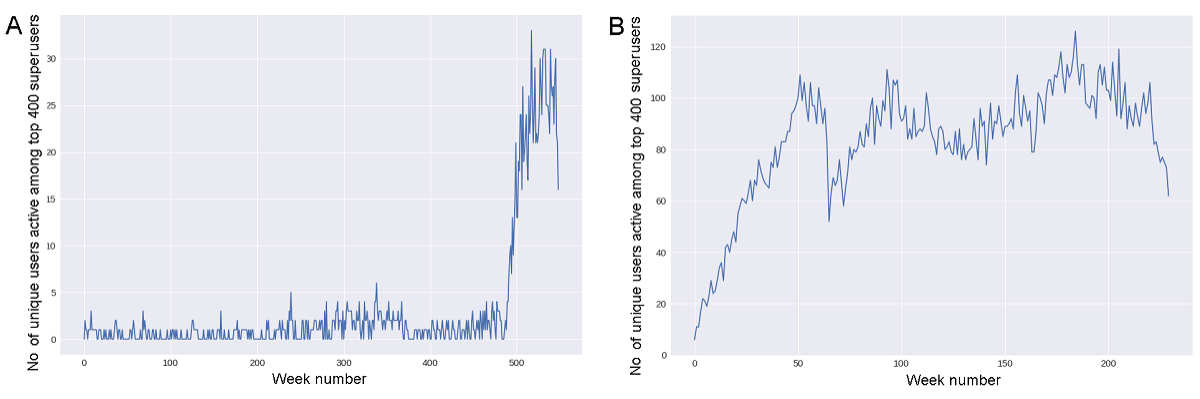
Number of unique users among the top 400 superusers as a function of time (weeks) within Asthma UK (A) and the British Lung Foundation (B).

Figure 8 suggests that there was no scarcity of superusers throughout the whole period of observation. In particular, the number of superusers in the Asthma UK community remained stable across almost the entire period until it increased substantially when the forum moved to the HealthUnlocked platform in 2015. Since then about twenty superusers have been active in the forum. On the other hand, in the BLF community the number of unique superusers increased steadily over the first 50 weeks (1 year) since inception (2015), and subsequently there were about 80-100 superusers regularly engaged with the community.

### Superusers’ Posting Activity

**Figure 9:**
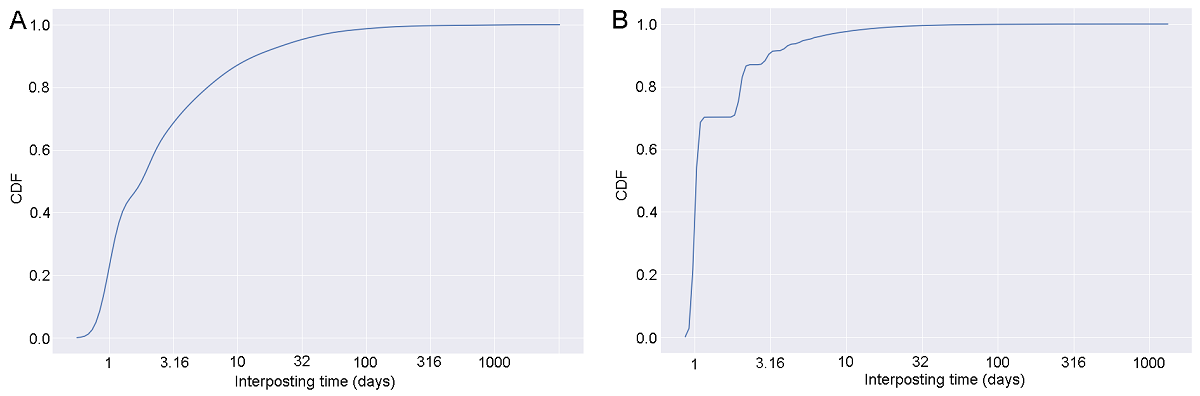
Cumulative distribution function (CDF) of the interposting time for the top 5% of users by post contribution within the Asthma UK (A) and the British Lung Foundation (B) communities.

We then investigated whether superusers’ posting activity was frequent and regular over time. To this end, for each of the top 5% users by post contribution, calculated cumulatively over the entire observation period, we measured the time interval separating every two subsequent posts to both communities. We then computed the inter-event time distributions for both communities to assess frequency and patterns of activity. Figure 9 suggests that 70% of interposting times were shorter than 3.1 days in the Asthma UK community, while 65% of interposting times in the BLF community were shorter than 1.7 days.

### Superusers’ Expertise

**Figure 10:**
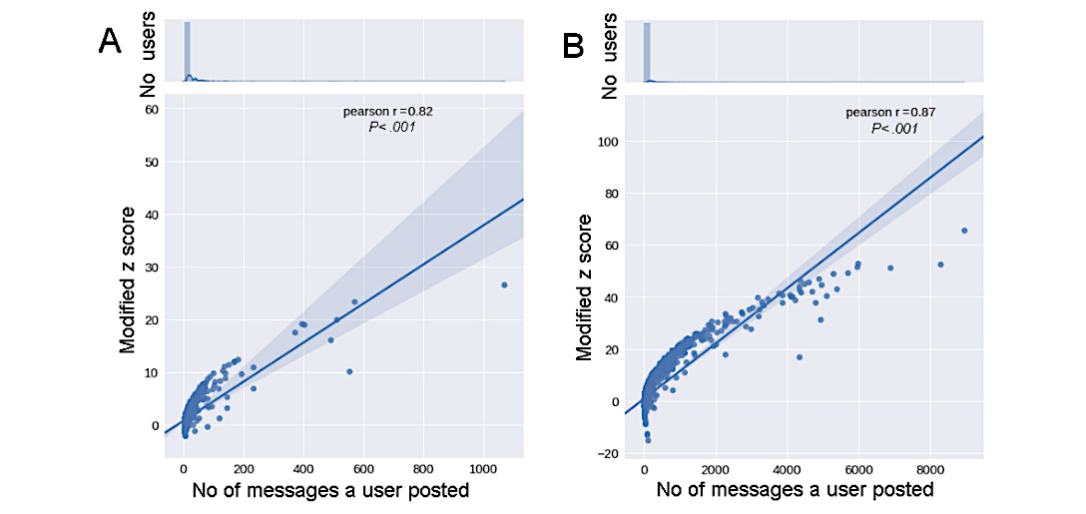
*Z*-score values of all users as a function of the number of posts written in the Asthma UK (A) and British Lung Foundation (B) communities. The top panels represent the normalized distributions of the number of users who wrote various numbers of posts.

For each user, a *z*-score was calculated in both communities to gauge the user’s expertise (see Data Analysis section). Figure 10 suggests that the more users became active in the communities, the more likely they were to write posts (assumed to be “help-giving” posts) [36,45] than to start new threads (assumed to be “help-seeking” posts). Such a finding might indicate that superusers were also those with the necessary degree of expertise to answer a large number of questions.

Thus, superusers not only play a topologically important role in the communities, but they are also likely to provide the expertise needed to answer queries.

### Ego Networks of Superusers

Next, we examine whether the ego networks of different types of users were topologically different, and what generated such differences. Users commonly started a discussion thread by writing a root post (ie, the post at level 1 of the thread). Several users could then directly respond to these posts at level 1, thus creating level-2 posts. More generally, according to the design of the communities, by posting a response to a level–(t) post, users created a level–(t+1) post. There was no limitation to how a post thread could evolve, and therefore to the complexity of the thread hierarchy. Information on post levels was made available through the post metadata. In our analysis, any post at level 2 or higher was classified as a level–2+ post. Here the analysis was restricted to the BLF forum, as the Asthma UK community was significantly smaller with simpler hierarchical levels.

**Figure 11:**
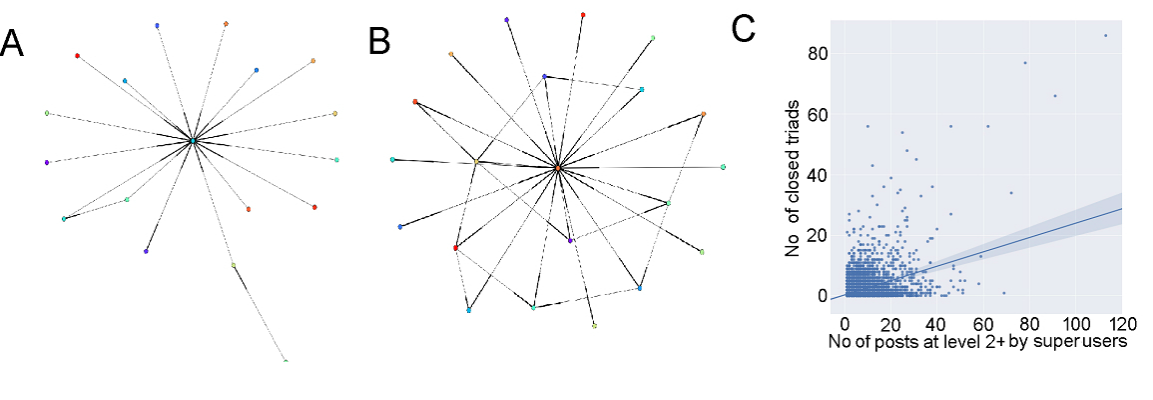
Topology of two illustrative ego networks created by a user with low (A) and high (B) posting activity in the British Lung Foundation community. Panel C shows the number of closed triads in ego networks as a function of posting activity of superusers (top 5% of users by post contribution).

Figure 10A and B show the ego networks of two types of users: one where the help-seeker, called root poster, contributed back multiple times to the thread itself, and the other where this pattern did not happen. In both cases, the thread received similar community engagements in terms of responses from other users. Figure 11B suggests that the highly active root poster developed a more cohesive network, rich in third-party relationships. In this ego network, many alters indeed connected with one another, thus creating closed triads centered on ego. In simple words, these users’ posting activity had the effect of making other users talk to each other, thus increasing integration and cohesion within the community. By contrast, the ego network developed by the root poster characterized by a lower contribution to the thread (Figure 11A) had a star-like shape and was rich in structural cleavages between alters. In this ego network, alters were disconnected from each other, and ego acted as the broker enabling indirect connections between alters. In simple words, these users did not favor connections between other users.

By replying to other users’ posts, superusers contributed significantly to level 2 or above. Figure 11C shows that there was a significant correlation between the number of triads in an ego network and the number of times ego (the root poster) contributed to the thread itself. The correlation coefficient between the number of triads and the number of posts at level 2 or above written by the top 5% of users by post contribution is 0.44 (*P*<.001).

When root posters responded back to the posts received, they created a more cohesive network structure. Most of these highly active users were superusers. This suggests that superusers, by posting “help-giving” posts, enabled other users to talk to each other, thus facilitating the formation of ties between them.

## Discussion

### Summary of Main Findings

In this study, we applied network analysis to two online communities for patients with chronic respiratory conditions to shed light on potential structural mechanisms underlying the role of these communities as scalable, peer-to-peer self-management support intervention systems. We found that the number of users and posts increased steadily over the years in the period of analysis. The majority of users were mutually reachable, either directly or indirectly, and formed a large connected component, which underlies the strength of the network as a means for widespread diffusion of information.

Superusers played a central role in these communities as a result of the characteristics of their posting activity and their constant online engagement. They preferentially replied to posts from peripheral users who were not equally well connected. In doing so, they additionally facilitated tie formation between users. Sensitivity analysis showed that gradual removal of superusers induced the network to collapse. Thus, superusers were responsible for holding the network together and, in particular, for ensuring the emergence of a large connected component. As a result, without superusers, there would be no effective spread of information within the community. Superusers acted as a continuously available resource over time. As users became more active within the community, they became more likely to reply to posts than to ask questions. This suggests that superusers gradually became “experts” providing others with advice and support, which is in agreement with what has recently been suggested by other qualitative studies [6,51].

### Strengths and Limitations

Based on social network analysis, this work has started elucidating crucial mechanisms underlying the potential of online health communities to promote effective self-management support interventions, in particular regarding the role of superusers in sustaining and providing integration and cohesion to the network. By analyzing the communities over more than five years, we have shown that superusers are a resource naturally present, able to sustain a network and make it thrive over time. This could prompt future studies to understand their role as a potential scalable health care workforce [1].

Limitations of this study include the lack of demographic and clinical information of participants as well as verification and validation of the information shared online [52], although previous qualitative work by the authors has identified Asthma UK superusers as adolescents with asthma [25]. Moreover, findings were not validated through the semantic analysis of the posts.

We did not investigate the reasons explaining the oscillating number of posts per user per week in the 2 communities, nor the time patterns of posting activity, nor the higher and regular number of posts of BLF users compared with Asthma UK ones. Time patterns of posting activity may reflect the nature of symptoms of the underlying lung conditions (see Figure 4). In particular, the uniformity of posting activity of BLF users might reflect daily self-management activities, whereas the time patterns uncovered for Asthma UK users might reflect self-management activities triggered by episodic exacerbations of symptoms.

More research is also needed to explore the mechanisms sustaining the effectiveness of health online communities and online engagement [53] in terms of the users’ quality of life and, more generally, the generation of beneficial health-related outcomes [54]. The role of superusers in the spread of information within online communities calls for further research to investigate how they can improve quality of information and reduce any potential harm [55]. Future work along these lines will integrate available evidence that incorrect or misleading information is, in many cases, efficiently corrected by peers [6,56]. Moreover, recent research has suggested that leveraging superusers to promote users’ online engagement may not achieve improved health-related outcomes, at least in connection with smoking cessation [57]. More qualitative work should, therefore, shed light on the role of superusers as actual providers of help and advice to other users.

Finally, 90% of people accessing patients’ online communities are passive readers who do not engage in online discussions [44,58]. This means that the number of registered users who post in the forum may represent only 10% of the people who access the community. However, how this large majority of patients that passively access patients’ online communities can benefit from reading others’ posts requires further investigation [59]. In particular, it remains unclear whether passive users can improve their self-management and other health-related behaviors, although previous work has shown that participation in online communities can increase passive users’ sense of belonging [60]. Change in behaviors of passive readers needs to be fully accounted for to examine the cost-effectiveness of peer-based online support interventions, compared with more traditional intervention tools. Moreover, it remains to be investigated whether there are variations in cost-effectiveness across active users and sub-groups of them with different patterns of social ties [61].

### Comparison With Related Work

Previous studies on medical online communities agree that users can benefit from the emotional support as well as the cumulative experiential information provided by others [1,62,63]. The value of online self-management support lies in the availability of co-created experiential knowledge and the presence of distributed health literacy. This enables users to find the information they require to manage their condition, and thus allows them to benefit from the health literacy of others in the network [1].

A qualitative study that was performed on a forum of people with stroke has shown that up to 95% of users’ intents for writing posts were met by replies [22]. In agreement with previous reports [45], we found that superusers represented a small proportion of the users in both communities, though they contributed to a considerable proportion of the overall posts. Superusers were members who assumed leadership roles by providing support, advice, and direction to other members [64,65].

This is in qualitative agreement with recent work on an online community for people with stroke, where superusers were shown to play an essential role in nurturing the ability of the forum to provide feedback and identify inappropriate information and health behaviors in the context of secondary prevention medications [6]. Interestingly, a related study using linguistic analysis showed that as users’ engagement in the community increased, their use of language changed. For example, it has been documented that the frequency of imperative verbs rose steadily through membership length, as superusers explicitly directed new members to do certain things [51].

Finally, superusers’ engagement with the online community and their daily commitment raise questions about what motivates their behavior. Recent work has suggested that their behavior can be motivated by perceived improvements in sense of well-being [4]. Thus, superusers can themselves profit from their engagement with online health communities. However, what remains to be investigated is whether and to what extent spending so much time in online health communities might be detrimental to superusers’ self-management.

### Implications for Policy, Practice, and Research

As a result of the voluntary basis of users' contributions, self-management support through online health communities offers high potential for cost-effectiveness from the perspective of formal services. Current health care challenges [66] include supporting self-care and management of LTCs. A key to future changes in models of health and social care are the expansion of health services offered locally as well an increasing role for patient self-management of LTCs. Initiatives to improve access to care in the community include expanding health care team to incorporate more allied health care professionals [67]. The benefits of self-management have not been realized through conventional face-to-face channels [18]. Could superusers represent an allied health care workforce, providing a means for health and social care integration? The impact and benefit of this novel approach could be huge and include: (a) increasing the confidence of a large number of people to self-care, (b) reducing demand on general practices [15], emergency care services and hospitals, and (c) saving money within health care systems, and across society as a whole. The potential scale of societal benefits would likely outweigh the opportunity costs associated with the time contributed by users. Understanding the mechanisms underlying effectiveness and uncovering how online communities are organized and evolve are vital preludes to developing and testing effective interventions and are required by the Medical Research Council Complex Interventions Framework [34]. However, little work has addressed this area to date. Although there is evidence that highly engaged users play a role as active help-providers to other users [45], this is to our knowledge the first study showing that superusers in online health communities: (1) are responsible for holding the community together, (2) engage with other users with low posting activity, and (3) indirectly contribute to tie formation between other users.

This work has drawn on social network analysis to uncover fundamental mechanisms underlying the potential of online communities to promote effective self-management support interventions. In particular, our study contributes to a better understanding of the role played by superusers in sustaining and providing integration and cohesion to the network. By analyzing the communities over more than five years, we have shown that superusers can sustain and make the network thrive over time. The presence of both a large connected component and superusers is a crucial feature of successful health communities. It is well known that components are critical for information diffusion [50,68]. Without a large connected component, users would be members of small isolated islands, and information would be unable to flow from one island to another. An online community needs a large component to function effectively. As edges between users are added over time, a large component is likely to emerge [69]. Our work has shown not only that superusers play a critical role in the emergence of a connected component, but also that, even without being “appointed” externally, superusers would emerge as the community grows large enough. Our findings will, therefore, prompt and inform future research interested in understanding superusers’ role as a potential scalable health care workforce in online self-management support interventions [1,70].

Moreover, our study has uncovered temporal patterns of posting activity. This will prompt further research aimed at investigating differences in these patterns across communities using qualitative analysis. This would include the analysis of whether users’ intents were met by replies [22] and the potential correlation between the amount of time spent online and improved disease self-management.

Across a variety of empirical domains, it has been documented that hubs (ie, nodes with a disproportionally large number of connections) are valuable resources that help spread information widely and amplify information cascades [71], help design effective vaccination campaigns and selective immunization strategies against disease diffusion and epidemics [72,73], and help improve the system’s robustness and vulnerability to random failures [74]. Here we have shown that health online communities are no exception. Our results suggest that superusers indeed represent a crucial resource for the integration and functioning of such communities, which therefore seem to be governed by the same network mechanisms as other real-world networks. This study will, therefore, inform future research interested in uncovering the common organizing principles underpinning a variety of real-world systems.

### Conclusions

This study shows that patients’ online communities share the same network features as other complex networks across a variety of empirical domains. Our analysis highlighted the special role played by superusers, their topological positions and behavior in the communities. In this sense, our results shed light on the topological mechanisms underlying the ability of patients’ online communities to provide self-management support and may, therefore, suggest levers for improving the quality of health care intervention.

At a time when health care services are working beyond capacity and patients are finding it difficult to access care, online communities provide the potential for addressing critical health care challenges. They offer a feasible way for patients with LTCs to find helpful advice and support, and a potentially cost-effective and scalable solution to the vast and rising costs associated with long-term disease management. Even though our results showed that there was no scarcity of superusers throughout the whole period of the study, nonetheless ensuring that such networks will become a core component of illness self-management on a broader scale requires proper research investment leading to randomized control studies and potentially a change in the concept of the health care team.

## Appendix

Multimedia Appendix 1 Asthma UK cumulative activity over the analysis time frame.

Multimedia Appendix 2 British Lung Foundation cumulative activity over the analysis time frame.

## Abbreviations

BLF: British Lung Foundation
COPD: chronic obstructive pulmonary disease
LTC: long-term condition
