# Addendum to the Acknowledgements: How Online Communities of People With Long-Term Conditions Function and Evolve: Network Analysis of the Structure and Dynamics of the Asthma UK and British Lung Foundation Online Communities

**DOI:** 10.2196/11564

**Published:** 2018-09-04

**Authors:** Sagar Joglekar, Nishanth Sastry, Neil S Coulson, Stephanie JC Taylor, Anita Patel, Robbie Duschinsky, Amrutha Anand, Matt Jameson Evans, Chris J Griffiths, Aziz Sheikh, Pietro Panzarasa, Anna De Simoni

**Affiliations:** ^1^ Department of Informatics King's College London London United Kingdom; ^2^ School of Medicine University of Nottingham Nottingham United Kingdom; ^3^ Asthma UK Centre for Applied Research Barts Institute of Population Health Sciences Queen Mary University of London London United Kingdom; ^4^ Primary Care Unit Department of Public Health and Primary Care University of Cambridge Cambridge United Kingdom; ^5^ HealthUnlocked London United Kingdom; ^6^ Asthma UK Centre for Applied Research Usher Institute of Population Sciences and Informatics University of Edinburgh Edinburgh United Kingdom; ^7^ School of Business and Management Queen Mary University of London London United Kingdom

The authors of “How Online Communities of People With Long-Term Conditions Function and Evolve: Network Analysis of the Structure and Dynamics of the Asthma UK and British Lung Foundation Online Communities” (J Med Internet Res 2018;20(7):e238) wish to add the following sentence to the Acknowledgments:

Nishanth Sastry was partially supported by the Grant No. ES/M00354X/1 (A Shared Space and a Space for Sharing: A Transdisciplinary Exploration of Online Trust and Empathy).

The correction will appear in the online version of the paper on the JMIR website on September 4, 2018, together with the publication of this correction notice. Because this was made after submission to PubMed, Pubmed Central, and other full-text repositories, the corrected article also has been re-submitted to those repositories.

